# Ten-Year Outcomes of Percutaneous Radiofrequency Ablation for Colorectal Cancer Liver Metastases in Perivascular vs. Non-Perivascular Locations: A Propensity-Score Matched Study

**DOI:** 10.3389/fonc.2020.553556

**Published:** 2020-10-16

**Authors:** Binbin Jiang, Hongjie Luo, Kun Yan, Zhongyi Zhang, Xiaoting Li, Wei Wu, Wei Yang, Minhua Chen

**Affiliations:** ^1^ Key Laboratory of Carcinogenesis and Translational Research (Ministry of Education/Beijing), Department of Ultrasound, Peking University Cancer Hospital & Institute, Beijing, China; ^2^ Department of Hepatobiliary, Pancreatic and Minimally Invasive Surgery, Zhengzhou Central Hospital Affiliated to Zhengzhou University, Zhengzhou, China; ^3^ Key Laboratory of Carcinogenesis and Translational Research (Ministry of Education/Beijing), Department of Radiology, Peking University Cancer Hospital & Institute, Beijing, China

**Keywords:** colorectal cancer, liver metastases, perivascular locations, radiofrequency ablation, treatment outcome

## Abstract

**Purpose:**

To compare long-term outcomes of percutaneous radiofrequency ablation for colorectal liver metastases in perivascular versus non-perivascular locations.

**Methods:**

This retrospective study included 388 consecutive patients with colorectal liver metastases (246 men, 142 women; age range 27–86 years) who underwent percutaneous radiofrequency ablation between January 2006 and December 2018. Propensity-score matching was performed for groups with perivascular and non-perivascular colorectal liver metastases. Rates of accumulative local tumor progression, overall survival, intra/extrahepatic recurrence, and complications were compared between the two groups.

**Results:**

We successfully matched 104 patients each in the perivascular and non-perivascular groups (mean age: 60.1 ± 11.5 and 60.1 ± 11.3 years, respectively). Cumulative local tumor progression rates at 6 months, 1 years, 3 years, and 5 years, respectively, were 8.8%,14.8%, 18.9%, and 18.9% in the perivascular group and 8.8%, 13.1%, 15.5%, and 15.5% in the non-perivascular group. The 1-, 3-, 5-, and 10-year overall survival rates, respectively, were 91.3%, 45.6%, 23.9%, and 18.7% in the perivascular group and 88.0%, 47.2%, 27.2%, and 22.6% in the non-perivascular group. No significant between-group differences were detected in cumulative local tumor progression (*p*=0.567, hazard ratio: 1.224) or overall survival (*p* = 0.801, hazard ratio: 1.047). The major complication rate was 1.0% (1/104, *p* > 0.999) in both groups. Tumor size was the only independent prognostic factor for local tumor progression (hazard ratio: 2.314; *p* = 0.002). On multivariate analysis for overall colorectal liver metastases, tumor diameter >3 cm, tumor location in the right colon, multiple tumors, and extrahepatic metastases before radiofrequency ablation (hazard ratios: 2.046, 1.920, 1.706, and 1.892, respectively; all *p* < 0.001) and intrahepatic recurrence (hazard ratio: 1.564; *p* = 0.002) were associated with poor overall survival.

**Conclusion:**

Cumulative local tumor progression, overall survival, and major complications rates did not differ significantly between perivascular and non-perivascular colorectal liver metastases after percutaneous radiofrequency ablation. For perivascular colorectal liver metastases, percutaneous radiofrequency ablation is a safe and effective treatment option.

## Introduction

The liver is the most frequent site of metastases from colorectal cancer (1), and surgical resection is a standard treatment for colorectal liver metastases (CLM). However, only 10–20% of patients with CLM are eligible for tumor resection due to high tumor burden and clinical complications (2). The National Comprehensive Cancer Network (NCCN)guidelines and European Society for Medical Oncology (ESMO) consensus guidelines recommended ablation as a local curative option for patients with metastatic colorectal cancer to the degree that all visible tumors can be eradicated (3, 4). Radiofrequency ablation (RFA) is an effective treatment in patients with CLM and can achieve high local control rates (5, 6). RFA finds widespread application for liver cancer due to its safety and low rate of major complications (1.3–7%) (6–8).

Tumor location close to the subcapsular region, diaphragm, gastrointestinal tract, and large blood vessels (9, 10) may be a key factor affecting ablation results because it may not permit a sufficient ablative margin and potentially influence tumor necrosis, resulting in high rates of local tumor progression (LTP). In addition, a randomized phase II trial study demonstrated that aggressive RFA treatment can prolong overall survival (OS) in patients with unresectable CLM (11).

The therapeutic outcome of RFA for liver tumors near large blood vessels remains controversial (12–15). A study reported (12) that perivascular location was a prognostic factor in patients with CLM who underwent RFA; perivascular location was associated with higher LTP rates, possibly attributable to the heat sink effect wherein blood flow dispels thermal energy away from the targeted tissue, leading to a reduced coagulation volume and an inadequate ablation margin (16). However, inconsistent conclusions have been reported in the literature; one study reported that RFA for CLM close to large hepatic vessels was safe and effective, perivascular location was not a risk factor for LTP (13). Furthermore, no guidelines are available for RFA for the treatment of perivascular CLM.

This study aimed to use propensity-score matching to compare the long-term outcomes of percutaneous RFA for perivascular and non-perivascular CLM and to identify the risk factors of patients with CLM underwent percutaneous RFA.

## Materials and Methods

### Patient Selection

**Figure 1:**
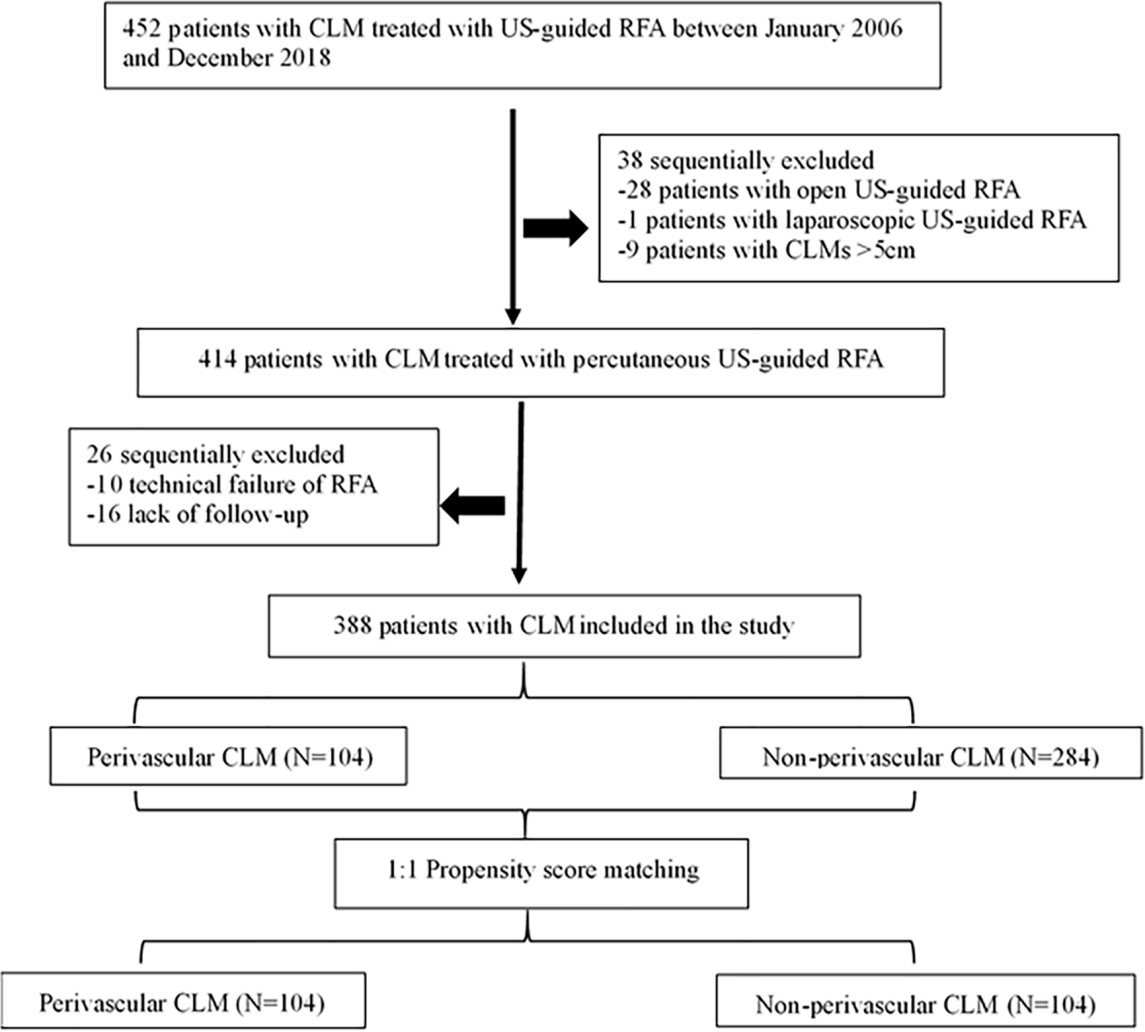
Flow diagram of patient selection for the study. CLM, colorectal liver metastases; US-guided, ultrasound-guided; RFA, radiofrequency ablation.

The institutional review board of the hospital approved this study, and the requirement for informed consent was waived because of the retrospective study design. Between January 2006 and December 2018, 452 patients with CLM were treated with ultrasound-guided RFA in our hospital. Of these, we identified 388 consecutive patients (mean age: 59.4 ± 11.0 years, range: 27–86) with CLM who underwent percutaneous RFA, either determined by a consensus of a multidisciplinary team or who refused surgery, were enrolled in the study. The eligibility criteria included: (a) tumor size ≤ 5 cm in diameter and the number of liver metastases ≤ 9; (b) conventional ultrasound or contrast-enhanced ultrasonography (CEUS) showing hepatic metastasis and treatment with percutaneous RFA under US-guidance; (c) absence of uncontrolled extrahepatic disease; (d) normal coagulation status and a liver function Child-Pugh A and B; (e) reported technical effectiveness of RFA; and (f) > 12-month follow-up. Exclusion criteria: (a) significant direct tumor invasion of adjacent organs or tumor thrombi in the main or lobar portal system; (b) the distance between the tumor and the first-level branch of the bile duct (common liver duct, left and right liver ducts) is ≤ 0.5cm; and (c) patients with serious diseases, such as congestive heart failure, myocardial infarction, and stroke in the past 6 months (**Figure 1**).

### Definition of Perivascular CML

In the absence of a standard definition, we defined a perivascular hepatic tumor as an index tumor having any contact with the first- or second-degree branches of a portal or hepatic vein (13, 15), with an axial diameter ≥ 3 mm (based on previous experimental and clinical studies) (17, 18).

If the index tumor was located near more than one large vessel, the largest vessel was selected as the reference vessel. Pre-treatment computed tomography (CT) or magnetic resonance imaging (MRI) results were reviewed by blinded radiologists with > 5 years of experience. All tumors were retrospectively categorized into the perivascular or non-perivascular group.

### RFA Procedure

Prior to RFA, all patients underwent US or enhanced US to assess the feasibility of US-guided percutaneous RFA. The treatment plan was determined by least three experts on RFA, according to the clinical conditions. All RFA procedures were conducted under real-time US guidance by four radiologists (CMH, YK, WW, and YW) who had > 10 years of experience in US-guided interventional procedures. For tumors abutting large vessels, treatment protocols were similar to the protocols that have been previously reported (19). All ablations were undertaken using the available RFA system: Celon Lab Power ablation system (Olympus, Germany); the Valleylab system (Tyco Healthcare, North Haven, CT); or the RITA Model 1500x ablation system (AngioDynamics, Latham, NY), according to the manufacturer’s instructions. Real-time ultrasound systems, Aloka ultrasound systems (Alokaα-10, Tokyo, Japan) or GE systems (E9, GE, United States), were used for scanning the lesion with 3.5–5.0 MHz convex probes and needle-guide devices for RFA procedures. As previously described (20), one physician located and guided the lesions in real time, while another inserted the electrode needle into the tumor. Most RFA devices can create an ablation sphere with a maximum diameter of 5 cm in the liver, but when the tumor diameter exceeds 3 cm, a strategy involving multiple overlapping ablations is employed (21). The post-RFA follow-up included routine tracking of the ablated lesions.

### Follow-Up and Outcomes

Within a month before performing RFA, enhanced CT or MRI and US of the abdomen were conducted. At 1month post-RFA, enhanced CT was performed to determine lesion persistence to evaluate the effectiveness of RFA. For follow-up, patients were examined with contrast-enhanced US, enhanced CT, or MRI every 3 months in the first 2 years after RFA and every 6 months thereafter. The following definitions used in our study are based on the standardization recommended by the International Working Group on Image-Guided Tumor Ablation (22). Technical effectiveness referred to the ablation area completely covering the tumor during the first enhanced imaging follow-up 1month post-RFA. LTP was defined as the appearance of new lesions at the edge of the ablation zones wherein the RFA had been technically effective. OS was calculated from the start of ablation treatment to death or the last follow-up. Intrahepatic recurrence was defined as a lesion with characteristics similar to those of the primary lesion but without contact with the original ablation zone in the liver. A major complication was an event that led to substantial morbidity and disability, increased the level of care, resulted in hospital admission, or lengthened hospital stay.

### Statistical Analysis

To reduce the effect of selection bias and baseline imbalances between the perivascular and non-perivascular groups, we performed propensity-score matching for the clinical characteristics of each group based on each patient’s propensity-score, which was estimated *via* logistic regression (23). The caliper value was 0.02 to performed propensity-score matching. Standardized mean differences of <0.10 indicated minute differences. Variables including age, sex, tumor size, primary location, T stage, lymph node metastases, time to liver metastases, number of liver metastases, history of resection for liver metastases pre-RFA, and extrahepatic metastases achieved the balance between the perivascular and non-perivascular groups after propensity-score matching.

The Wilcoxon rank sum test or independent *t*-test was used for continuous variables, and the chi-square test or Fisher exact test was used for categorical variables. The rates of LTP, OS, and intrahepatic and extrahepatic recurrence were estimated by the Kaplan–Meier method with the log-rank test. Univariate and multivariate analyses of all data were carried out using a Cox proportional hazards regression model for LTP and OS. Statistical analyses were conducted using SPSS 22.0 (SPSS Inc., Chicago, IL) and R version 2.15.x (R Foundation for Statistical Computing, Vienna, Austria). Differences with a *p* value < 0.05 were considered statistically significant.

## Results

### Baseline Characteristics

Baseline characteristics of all CLM patients (n = 388; mean age: 59.4 ± 11.0 years, range: 27–86) and lesions (n = 388; mean size: 2.4 ± 1.0 cm, range: 0.6–4.9 cm) are presented in **Table 1**. The median follow-up period was 45.0 (range: 0–161) months for CLM. At the first enhanced imaging follow-up that was performed 1-month post-RFA, the rate of technical effectiveness was 97.6% (404/414) for CLM treated with RFA. The perivascular group showed higher proportions of primary left colon lesions (88.5% vs. 78.9%; *p* = 0.031) and male patients (72.1% vs. 60.2%; *p* = 0.031) than the non-perivascular group. The baseline characteristics were well balanced between the two groups (**Table 1**).

**Table 1:** Demographic and clinical characteristics of patients with colorectal liver metastases (CLM).

Variable	Perivascular	Before Matching			After Matching		
		Non-perivascular	*p*	St.MD	Non-perivascular	*p*	St.MD
	(n = 104)	(n =284)	Value		(n = 104)	Value	
Age at enrollment (year)*	60.14 ± 11.51	59.14 ± 10.86	0.427	0.091	60.11 ± 11.25	0.981	0.060
No. of men	75(72.1)	171(60.2)	0.031	0.264	75(72.1)	1.000	0.000
Tumor size (cm)^+^	2.3(1.8–3.2)	2.2(1.6-3.0)	0.157	0.111	2.4(1.8–3.2)	0.827	0.043
≤3cm	75(72.1)	219(77.1)	0.309			73(70.2)	0.760	
>3cm	29(27.9)	65(22.9)				31(29.8)		
Primary location			0.031	0.299		1.000	0.000
Right colon	12(11.5)	60(21.1)				12(11.5)		
Left colon	92(88.5)	224(78.9)				92(88.5)		
T3-4 stage	99(95.2)	272(95.8)	0.783	0.027	98(94.2)	0.757	0.045
Lymph node metastasis	82(78.8)	204(71.8)	0.164	0.171	84(80.8)	0.730	0.047
Synchronous liver metastasis	57(54.8)	132(46.5)	0.146	0.167	52(50.0)	0.488	0.096
No. of liver metastases			0.437	0.089		0.576	0.077
Single	47(45.2)	141(49.6)				43(41.3)		
Multiple	57(54.8)	143(50.4)				61(58.7)		
Liver metastases resection pre-RFA	39(37.5)	113(39.8)	0.682	0.047	41(39.4)	0.776	0.040
Extrahepatic metastases pre-RFA	34(32.7)	100(35.2)	0.644	0.053	38(36.5)	0.560	0.082
Type of peritumoral vessel								
Portal vein	52(50.0)							
Hepatic vein	52(50.0)							

Unless indicated otherwise, data are the number of patients, with percentages in parentheses. Values of standardized mean differences less than 0.10 indicate better balance.

*Data are means ± standard deviations, were analyzed using the two-sample t test.

^+^Data are medians, with interquartile ranges in parentheses, were analyzed using Wilcoxon rank sum test.

The categorical variables were analyzed using the x^2^ test or Fisher exact test.

CLM, colorectal liver metastases; RFA, radiofrequency ablation; No. of liver metastases, number of liver metastases; St.MD, Standardized mean difference.

### Comparison of Outcomes Before Propensity-Score Matching

#### LTP and OS

During follow-up, LTP occurred in 18 of 104 patients (17.3%) in the perivascular group and 42 of 284 patients (14.8%) in the non-perivascular group (*p* = 0.543). Moreover, 38.9% (7/18) of patients with LTP were treated with RFA and 44.4% (8/18) underwent chemotherapy due to multiple or recurrent lesions. The cumulative LTP rates at 6 months, 1 years, 3 years, and 5 years were 8.8%, 14.8%, 18.9%, and 18.9% in the perivascular group and 6.9%, 11.2%, 19.7%, and 21.4% in the non-perivascular groups, respectively (*p* = 0.823). As of July 31, 2019, 70 of 104 (67.3%) patients in the perivascular group and 137 of 284 (48.2%) patients in the non-perivascular group had died. The 1-, 3-, 5-, and 10-year OS rates were 91.3%, 45.6%, 23.9%, and 18.7% in the perivascular group and 85.0%, 51.9%, 25.6% and 21.3% in the non-perivascular group (*p* = 0.798).

#### Intrahepatic and Extrahepatic Recurrence

In the perivascular and non-perivascular groups, 57 of 104 (54.8%) patients and 128 of 284 (45.1%) patients, respectively, had intrahepatic recurrence (*p* = 0.089); 60–70% of patients with intrahepatic recurrence received chemotherapy. The 1-, 3-, 5-, and 10-year intrahepatic recurrence rates were 33.3%, 56.8%, 60.1%, and 76.1% in the perivascular group and 32.8%, 55.6%, 59.0%, and 72.7% in the non-perivascular group (*p* = 0.705). Extrahepatic recurrence was identified in 49 patients (47.1%) in the perivascular group and 117 patients (41.2%) in the non-perivascular group. The 1-, 3-, 5-, and 10-year cumulative rates of extrahepatic metastases were 26.8%, 48.8%, 55.6%, and 60.1% in the perivascular group and 24.5%, 49.2%, 61.6%, and 65.5% in the non-perivascular group (*p* = 0.962).

#### Complications

Six (1.5%) major complications occurred in 388 patients within 30 days of RFA, as summarized in **Table 2**. There was one RFA-related death (0.3%) in an 84-year-old man with a history of cerebral hemorrhage and diabetes and a 4.6-cm tumor situated close to the hepatic vein. The patient developed abdominal hemorrhage and biliary effusion 3 days after RFA. Despite active treatment, the patient eventually died of septic shock 9 days after RFA. One of the three patients with liver abscess and one patient with pleural effusion were treated with percutaneous catheterization drainage; the other patients showed improvement with symptomatic treatment. The rate of major complications was 1.0% (1 of 104 patients) in the perivascular group and 1.8% (5 of 284 patients) in the non-perivascular group, with no significant intergroup difference (*p* > 0.999; **Table 2**).

**Table 2:** Incidence of major complications.

Major complications	Overall Data^*^		Matched Data^+^	
	Perivascular (n=104)	Non-perivascular (n=284)	Perivascular (n=104)	Non-perivascular (n=104)
Major Complications	1(1.0)	5(1.9)	1(1.0)	1(1.0)
Hepatic abscess	0	3(1.1)	0	1(1.0)
Acute cholecystitis	0	1(0.4)	0	0
Pleural effusion requiring drainage	1(1.0)	0	1(1.0)	0
Liver rupture	0	1(0.4)	0	0
Tumor seeding	0	0	0	0
Treatment-related death	1	0	1	0

data are the number of patients, with percentages in parentheses.

^*^p > .999; ^+^p > .999.

p obtained by using Fisher exact test.

#### Comparison of Therapeutic Outcomes After Propensity-Score Matching

**Figure 2:**
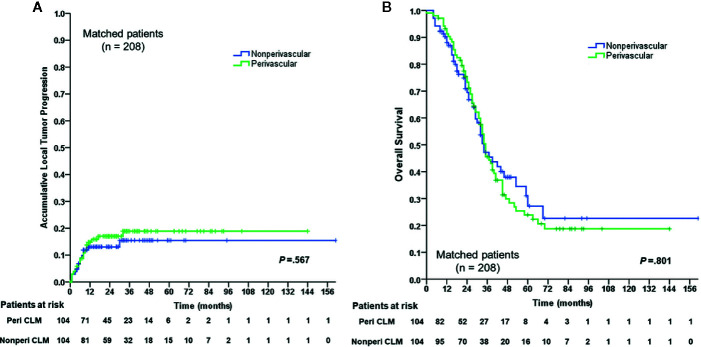
Cumulative LTP)rate and OS rate curves for the perivascular CLM and the non-perivascular CLM in matched data. **(A)** Cumulative local tumor progression in matched data. **(B)** Overall survival in matched data. The local tumor progression and overall survival were estimated by the Kaplan-Meier method with the log-rank test. LTP, local tumor progression; OS, overall survival; Peri CLM, perivascular colorectal liver metastases.

In the matched cohort, 104 perivascular CLM patients were all enrolled after propensity-score matching. In the non-perivascular group, LTP occurred in 14 of 104 patients (13.5%, *p* = 0.442; **Table 3**). The subsequent treatment modalities for patients are shown in **Table 3**. The cumulative LTP rates at 6 months, 1 year, 3 years, and 5 years were 8.8%, 13.1%, 15.5%, and 15.5%, respectively (*p* = 0.567; **Figure 2**); 51 of 104 (49.0%) patients died. The 1-, 3-, 5-, and 10-year OS rates were 88.0%, 47.2%, 27.2%, and 22.6%, respectively (*p* = 0.801; **Figure 2**), without significant differences in LTP and OS rates between the perivascular and non-perivascular groups.

**Table 3:** treatment modalities for patients with local tumor progression (LTP) and intrahepatic recurrence in matched groups.

Treatment Modalities	Local Tumor Progression^*^		Intrahepatic Recurrence^+^	
	Perivascula r (n=18)	Non-perivascular (n=14)	Perivascular (n=57)	Non-perivascular (n=46)
Resection	2(11.1)	4(28.6)	4(7.0)	4(8.7)
RFA	7(38.9)	5(35.7)	3(5.3)	3(6.5)
Radiotherapy	1(5.6)	1(7.1)	2(3.5)	3(6.5)
Resection + radiotherapy	0	0	1(1.8)	0
TACE	0	0	3(5.3)	0
RFA+TACE	0	0	1(1.8)	0
Gamma Knife Treatment	0	0	2(3.5)	0
Chemotherapy	8(44.4)	4(28.6)	39(68.4)	35(76.1)
Best supportive care	0	0	2(3.5)	1(2.2)

Data are the number of patients, with percentages in parentheses.

^*^p value between two groups was.442, ^+^p value between two groups was .127.

p value obtained by using the xχ^2^ test.

RFA, radiofrequency ablation; TACE, transcatheter arterial chemoembolization.

In the non-perivascular group, 46 of 104 CLM patients (44.2%) showed intrahepatic recurrence (*p* = 0.127, **Table 3**). The subsequent treatment modalities for patients with intrahepatic recurrence in both groups are shown in **Table 3**. The 1-, 3-, 5-, and 10-year intrahepatic recurrence rates were 30.2%, 56.3%, 59.4%, and 59.4%, respectively (*p* = 0.589). Moreover, 40.4% (42/104) of CLM patients showed extrahepatic recurrence during follow-up. The 1-, 3-, 5-, and 10-year extrahepatic recurrence rates were 22.8%, 47.4%, 58.9%, and 58.9%, respectively (*p* = 0.830). The rate of major complications was 1.0% (1 of 104 patients; *p* > 0.999; **Table 2**) in both groups.

### Analysis of Risk Factors Associated With Outcomes

The 6-month, 1-year, 3-year, and 5-year cumulative LTP rates were 7.4%, 12.2%, 19.3%, and 20.3%, respectively. The 1-, 3-, 5-, and 10-year OS rates were 86.7%, 49.5%, 25.2%, and 20.4%, respectively, for the overall CLM patients in the study. Multivariate analysis of all patients (n = 388), the results of which were expressed as hazard ratios (HRs) and 95% confidence intervals (CIs), showed that tumor size was an independent prognostic factor for LTP (HR: 2.314, 95% CI: 1.354–3.955, *p* = 0.002) (**Table 4**). In addition, tumor size (HR: 2.046, 95% CI: 1.511–2.769, *p* < 0.001), primary tumor location (HR: 1.920, 95% CI: 1.348–2.733, *p* < 0.001), number of liver metastases (HR: 1.706, 95% CI: 1.265–2.300, *p* < 0.001), extrahepatic metastases pre-RFA (HR: 1.892, 95% CI: 1.413–2.533, *p* < 0.001), and intrahepatic recurrence (HR: 1.564, 95% CI: 1.171–2.088, *p* = 0.002) were independent prognostic factors for OS in patients with CLM (**Table 4**).

**Table 4:** Univariable and multivariable analyses of prognostic factors for local tumor progression (LTP) and overall survival (OS) for overall colorectal liver metastases (CLMs).

Variable	Local tumor progression				Overall survival			
	Univariate analysis		Multivariate analysis		Univariate analysis		Multivariate analysis	
	HR (95%CI)	*p*	HR (95%CI)	*p*	HR (95%CI)	*p*	HR (95%CI)	*p*
Age (yr)	1.527(0.911–2.559)	.109	1.447(0.860–2.435)	.164	1.241(0.932–1.652)	.139	1.131(0.841–1.521)	.415
Tumor size (cm)	2.230(1.324–3.756)	.003	2.314(1.354–3.955)	.002	1.831(1.370–2.446)	<.001	2.046(1.511–2.769)	<.001
Sex	0.888(0.522–1.509)	.660			0.886(0.668–1.175)	.402		
Primary location	0.908(0.472–1.748)	.773			1.647(1.179–2.302)	.003	1.920(1.348–2.733)	<.001
T stage	0.994(0.311–3.177)	.992			1.763(0.829–3.748)	.141	1.351(0.614–2.972)	.454
Lymph node metastasis	0.649(0.384–1.098)	.107	0.627(0.361–1.091)	.098	1.887(1.326–2.685)	<.001	1.352(0.917–1.991)	.127
Synchronous liver metastasis	1.385(0.829–2.314)	.214			1.126(0.867–1.479)	0.396		
No. of liver metastases	0.600(0.357–1.011)	.055	0.692(0.403–1.187)	0.181	1.882(1.419–2.497)	<.001	1.706(1.265–2.300)	<.001
Liver metastasis resection pre-RFA	1.384(0.803–2.386)	.242			0.918(0.691–1.219)	.555		
Extrahepatic metastases	1.150(0.661–2.001)	.620			1.942(1.462–2.579)	<.001	1.892(1.413–2.533)	<.001
Perivascular location	1.065(0.612–1.851)	.825			1.038(0.778–1.386)	.800		
Intrahepatic recurrence	1.033(0.623–1.714)	.900			1.688(1.275–2.236)	<.001	1.564(1.171–2.088)	0.002
Extrahepatic recurrence	1.224(0.730–2.052)	.444			0.828(0.630–1.088)	.175		
LTP	–	–	–		1.053(0.746–1.488)	.768		

Data in parentheses are 95% confidence intervals. The Cox proportional hazards regression model was used for the univariable and multivariable analysis. Variables with p<0.15 in univariable analyses were included in the multivariable model.

LTP, local tumor progression; OS, overall survival; RFA, radiofrequency ablation; CLM, colorectal liver metastases; No. of liver metastases, number of liver metastases; HR, hazard ratio; CI, confidence interval.

### Subgroup Analysis for the Type of Peritumoral Vessels

**Figure 3:**
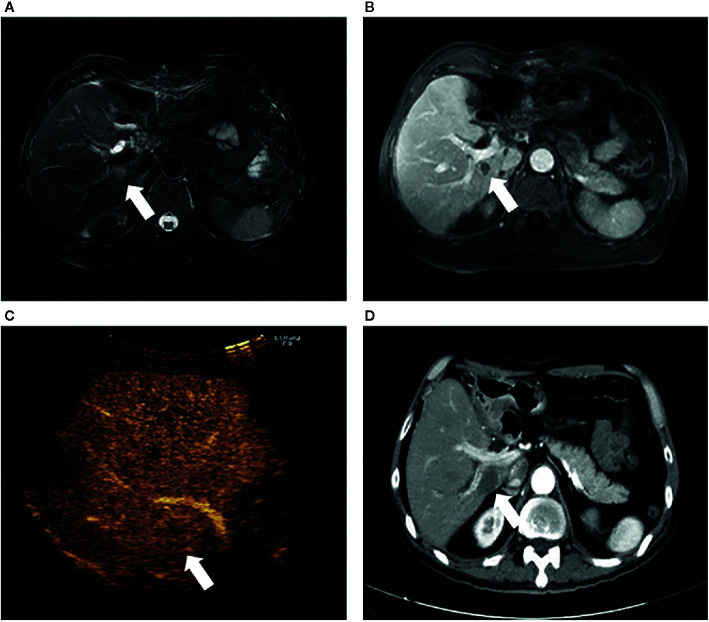
Images in a 61-year-old-man who underwent radiofrequency ablation (RFA) for periportal CLM. **(A)** Axial MRI T2-weighted images shows a 2.2-cm lesion of high signal intensity (arrow) in segment VII before RFA. **(B)** Axial enhanced MRI image shows that the lesion (arrow) washes out in equilibrium phase; **(C)** contrast-enhanced ultrasonography (CEUS) image before RFA shows that the index tumor (arrow) is in contact with the portal vein. The patient underwent RFA, and obtained technical effectiveness 1 month after RFA. **(D)** Axial enhanced CT image shows no local tumor progression around the ablation zone 17 months after RFA. CLM, colorectal liver metastases; US, ultrasound; RFA, radiofrequency ablation.

**Figure 4:**
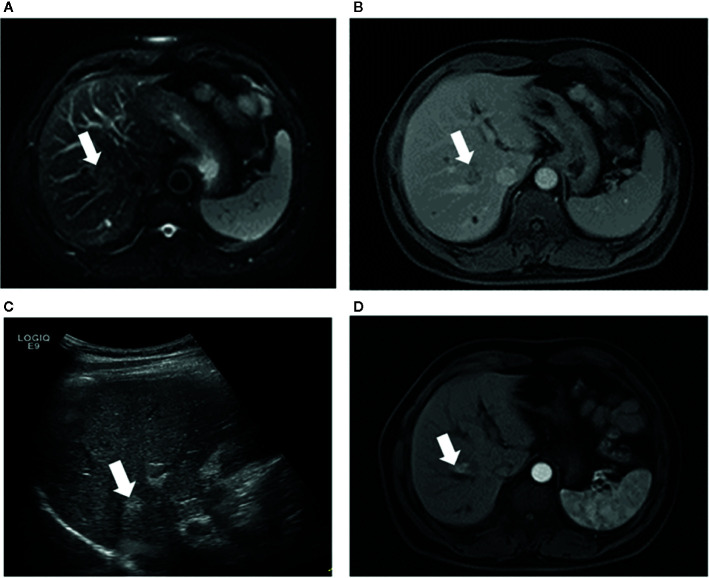
Images in a 53-year-old-man who underwent RFA for perihepatic CLM. **(A)** Axial MRI T2-weighted images shows a 2.6-cm lesion of high signal intensity (arrow) in segment VIII before RFA. The index tumor abuts the hepatic vein. **(B)** Axial enhanced MRI image shows that the lesion (arrow) washes out in equilibrium phase; **(C)** US image before RFA shows that the index tumor (arrow) is in contact with the hepatic vein. The patient underwent RFA, obtained technical effectiveness 1 month after RFA. **(D)** Axial enhanced MRI image showed local tumor progression around the ablation zone 6 month after RFA. CLM, colorectal liver metastases; US, ultrasound; RFA, radiofrequency ablation.

The type of peritumoral vessels was classified as periportal and perihepatic vessels in 52 (50.0%) and 52 (50.0%) patients, respectively (**Figures 3**, **4**). Furthermore, 7 of 52 patients (13.5%) and 11 of 52 patients (21.2%) in the periportal and perihepatic groups, respectively, showed LTP. The 6-month, 1-year, 3-year, and 5-year cumulative LTP rates were 5.8%, 9.8%, 14.1% and 14.1%, respectively, in the periportal group and 11.9%, 19.9%,23.4%, and 23.4%, respectively, in the perihepatic group (*p* = 0.285). In both groups, 35 of 52 patients (67.3%) with CLM died. The OS rates at 1, 3, 5, and 10 years were 92.2%, 43.4%, 22.5%, and 19.3%, respectively, in the periportal group and 90.3%, 47.6%, 24.8%, and 17.7%, respectively, in the perihepatic group (*p* = 0.920). The differences in LTP and OS between the periportal and perihepatic groups were not significant.

## Discussion

We identified that patients with CLM who underwent percutaneous RFA had similar rates of cumulative LTP, OS, and major complications in the perivascular and non-perivascular groups, both before and after propensity-score matching. This indicates that percutaneous RFA can be safe and effective for perivascular CLM.

Surgical resection is a standard treatment for patients with CLM (4), RFA cannot completely replace surgery, because of the low progression-free survival for lesions > 3cm (24). However, ESMO guidelines (3) recommend RFA as a curative option to eradicate all visible liver tumors, for patients with resectable lesions located deep in the liver where surgical resection would lead to a great loss of liver volume, with recurrence of lesions after liver surgical resection, for patients who are intolerant to surgery (advanced age, associated co-morbidity), and for patients who refused surgery. In this study, RFA demonstrated its safety in lesions adjacent to blood vessels.

It is generally believed that an inadequate ablation margin is an independent predictor of LTP after ablation for CLM (25–28). Investigators in previous studies have suggested (12, 14, 17) that perivascular location of a liver tumor was a risk factor for LTP after RFA, because of the inability to achieve an adequate margin in such cases, as blood flow dispels thermal energy away from the lesions. However, some studies (13, 15) showed that perivascular location should not be considered a risk factor for LTP after RFA. In our study, perivascular location was defined with respect to two criteria:1) the vessel diameter ≥ 3 mm. The appropriate cut-off of 3mm was based on the results of previously published animal experiments and clinical research (16, 17, 29–31), which showed an inverse correlation between vessel diameter and the degree of heat sink effect. Sink effect may occur over 3 mm in vessel diameter and cause incomplete ablation; and 2) any contact with first- or second-degree branches of a portal or hepatic vein based on CT/MRI. The latter criterion was accepted by most clinical studies (15, 29–32). The cumulative LTP rates after RFA were not significantly different between perivascular CLM and non-perivascular CLM patients in line with the previous studies (13, 15).

There are some possible reasons for the similar outcomes in the perivascular and non-perivascular groups: firstly, the equipment used for multipolar RFA (13, 33) for perivascular liver tumors has gradually improved, resulting in better local tumor control. Second, the “supplementary ablation,” “accumulative multiple ablations,” (34) and “multi-step ablation” (35) techniques are helpful in achieving local tumor control. Furthermore, physician expertise and experience facilitate successful ablation.

Previous studies (36) have demonstrated that insufficient RFA enhanced the metastatic ability of tumor cells, which was mediated by signaling and dissemination of cancer cells, leading to recurrence. However, there were no significant differences in intrahepatic and extrahepatic recurrence rates after RFA between the perivascular and non-perivascular groups. Therefore, this indicated that RFA techniques may offer sufficient ablation for both perivascular and non-perivascular CLMs. The effect of RFA on perivascular CLM was similar to that on non-perivascular CLM.

The zone of ablation is larger near the hepatic vein than near the portal vein because of different flow velocity (37); patients with tumors located near the main portal vein branch are at risk for rapid tumor progression after RFA (38). However, we found no significant differences between the periportal vessel and perihepatic vessel groups, which suggests that improved treatment strategies have a greater influence on the planning of the RFA target volume than the heat sink effect.

Previous studies have reported (6, 39) that 5-year OS rates ranged from 21% to 31% in patients with CLM treated with RFA; the LTP rates were in the range of 9–42% (40). We found similar outcomes in CLM patients treated by RFA. In this study, tumor size was the only independent prognostic factor for LTP. In addition, several prognostic factors of poor OS were identified: a tumor diameter > 3 cm, tumor location in the right colon, multiple tumors, extrahepatic metastases pre-RFA, and intrahepatic recurrence. These concur with previously reported prognostic factors (6, 13, 41–43), except for intrahepatic recurrence; this may be the reason that these investigators did not conduct further analysis into the relationship between intrahepatic recurrence and OS. However, intrahepatic recurrence may indicate the presence of more tumor cells in the blood, resulting in poor OS.

Complication rates between patients with perivascular and non-perivascular CLM treated with percutaneous RFA did not differ before or after propensity-score matching, which is consistent with previous results (15). Percutaneous RFA did not increase biliary complications, even when periportal tumors were possibly close to biliary duct structures in our study. This was because of the strict enrollment criteria and operating procedures. If the distance between the tumor and the first-level branch of the bile duct (common hepatic duct, left and right hepatic ducts) was ≤ 0.5 cm, patients did not meet the inclusion criteria for treatment with RFA. If a safe margin and a needle access route could possibly be obtained, patients were considered for treatment with RFA, and real-time ultrasound guidance was required during RFA to ensure that there was no damage to the bile duct. Therefore, perivascular CLM can be safely treated with RFA. Although there was one treatment-related death in this study, this patient had multiple RFA-related risk factors, including older age, multiple comorbidities, a large tumor, and a problematic tumor location. Hence, indications should always be evaluated carefully before RFA and treated prudently.

This study had several limitations. First, it was retrospective study. Although we conducted a propensity-score matched analysis to balance the baseline characteristics of patients, we cannot exclude the possibility of bias in terms of other confounding factors, such as the experience of the physician. Second, we failed to consider that other problematic tumor locations, such as locations close to the liver surface or the diaphragm, may influence the outcome of ablation. However, a study (34) reported that individualized treatment strategies can ensure that patients with problematic locations achieve outcomes similar to those of patients with non-problematic tumor locations. Finally, there is no universal consensus on the definition of perivascular tumors. Our definition of a perivascular tumor was consistent with that used in previous reports (13, 15, 17); however, this needs validation in future studies.

In conclusion, there were no significant differences in the rates of cumulative LTP, OS, and major complications between patients with perivascular CLM and non-perivascular CLM treated with percutaneous RFA. Thus, the findings provide evidence-based medical evidence that percutaneous RFA is a safe and effective treatment option for perivascular CLM.

## Data Availability Statement

The raw data supporting the conclusions of this article will be made available by the authors, without undue reservation.

## Ethics Statement

The studies involving human participants were reviewed and approved by the Research Ethics Committee of the Peking University Cancer Hospital. Written informed consent for participation was not required for this study in accordance with the national legislation and the institutional requirements.

## Author Contributions

KY, BJ, and HL conceived and designed the experiments. KY, ZZ, WW, WY, and MC performed clinical studies. BJ, HL, ZZ, and XL performed the data analysis and statistical analysis. BJ, HL, KY, and ZZ edited manuscript. All authors contributed to the article and approved the submitted version.

## Funding

This research was supported by Beijing Municipal Science and Technology Commission (grant no. Z151100004015186) and The capital health research and development of special (grant no.2020-2-2152).

## Conflict of Interest

The authors declare that the research was conducted in the absence of any commercial or financial relationships that could be construed as a potential conflict of interest.
